# Can radiographic plain film be used to determine the depth of the tumour bed in the absence of surgical clips for breast boost planning

**DOI:** 10.2349/biij.5.3.e11

**Published:** 2009-07-01

**Authors:** I Chitapanarux, M Muttarak, W Na-Chiangmai, H Trakultivakorn, A Somwangprasert, P Kamnerdsupaphon, E Tharavichitkul, V Sukthomya, V Lorvidhaya, A Watcharawipha

**Affiliations:** 1 Department of Radiology, Faculty of Medicine, Chiang Mai University, Thailand; 2 Department of Surgery, Faculty of Medicine, Chiang Mai University, Thailand

**Keywords:** absence of surgical clips, breast boost, radiographic film

## Abstract

**Purpose:**

A number of studies have demonstrated the importance of using surgical clips to define the tumour bed in breast boost radiotherapy. In the absence of such clips, other techniques suggested to improve boost location have included CT and ultrasound (US). Determination of the depth of the tumour bed is important in the selection of electron energy. This study was conducted to prospectively compare the depth of the lumpectomy cavity as defined by ultrasound to radiographic plain film evaluation of the anterior border of the pectoralis muscle.

**Materials and Methods:**

Forty-one breast-cancer patients treated at the Division of Therapeutic Radiology and Oncology, Department of Radiology, Faculty of Medicine, Chiang Mai University between December 2004 and December 2006 were prospectively identified as having no surgical clips within the lumpectomy cavity. All patients underwent both US evaluation of the depth of tumour bed (D1) and radiographic evaluation of the depth of the anterior border of the pectoralis muscle (D2). These depth dimensions (D1 and D2) were compared using a paired t-test. The correlation of both methods was analyzed by Pearson correlation test.

**Results:**

Depth dimensions by US were shorter than the radiographic film method in 85% of patients. The absolute mean difference of the depth (radiographic films minus US) was 0.129 cm. A paired t-test demonstrated that the difference between these two methods to be not statistically significant (p= 0.27). The absolute difference of depth between the two methods ranged from 0 to 0.5 cm. A significant correlation was found between US and radiographic film measurements (p<0.01).

**Conclusion:**

Plane radiographic film evaluation of the anterior border of the pectoralis muscle can be used to define the depth of the tumour bed in patients who have no surgical clips. However, the plane radiographic film method determines only the depth, not the transverse and longitudinal dimensions of the tumour bed. Additional information from US is needed to delineate the target volume for the tumour bed boost. In the absence of surgical clips, the authors recommend integration of both methods in breast boost planning process.

## INTRODUCTION

Breast conserving surgery followed by external beam radiotherapy is one standard treatment of early breast cancer. An additional radiation boost dose to the tumour bed or lumpectomy cavity is required after breast conserving surgery to reduce the risk of local recurrence. There are many ways to design the boost fields but no standard technique has been established. In a recent Patterns of Care Study on early stage breast cancer, the breast volume was determined by CT in 11.7%, a clinical fluoroscopic simulator in 43.9%, and clinically alone in 37.2% [1].

One of the most common planning employs clinical information and surgical scars. However, many reports have demonstrated the unreliability of this boost technique with geographic miss rate ranging from 43-68% [2-4]. The gold standard tool to guide the design of boost field is radiographic evaluation of intra-operatively placed surgical clips. However, placement of surgical clips is not routinely performed by all surgeons.

In the absence of surgical clips, other techniques suggested to improve boost location include computed tomography (CT) planning and ultrasound (US) [5-7]. Several studies of boost fields using US to identify the location and dimensions of the lumpectomy cavity found the fields were acceptable in size and that accuracy compared favourably to other techniques for boost planning [3-10]. However, Rabinovitch et al. recommended against using US for breast boost planning as it could result in inappropriate selection of low electron energies and small field sizes compared to the radiographic evaluation of surgical clips [11]. Similarly, CT can be used to target the tumour bed with cavity visualization similar to US [12], but CT is not available in all radiation centres.

The authors are seeking ways to shorten the process of defining the radiation boost plan in order to improve working practice at the Division of Therapeutic Radiology and Oncology where 200 patients were treated daily. Focusing on patients who had no clip placement, the authors conducted this study to compare the depth of the lumpectomy cavity as defined by ultrasound technique to radiographic plain film evaluation of the anterior border of the pectoralis muscle. The authors wished also to explore these relatively easy techniques of delineating the surgical bed for the benefit of institutions where other advanced techniques are not feasible. This study was approved by the Institutional Review Board of the Faculty of Medicine, Chiang Mai University.

## MATERIALS AND METHODS

Between December 2004 and December 2006, 54 patients underwent excisional biopsy and axillary lymph node dissection followed by irradiation at the Division of Therapeutic Radiology and Oncology, Department of Radiology, Faculty of Medicine, Chiang Mai University. The major inclusion criteria were 1) stage I and II breast cancer, 2) negative resection margins and 3) no surgical clip placement in the tumour bed. Nine patients with surgical clips and four patients with positive surgical margins were excluded. Thus, 41 patients were included in this study.

Before the day of initial simulation, all patients underwent US evaluation of the lumpectomy cavity to define all dimensions of the cavity. Dimensions of the cavity included transverse measurement (T) (medial to lateral), longitudinal measurement (L) (superior to inferior) and depth (D_1_) (skin to the posterior portion of the cavity). The extent of the lumpectomy cavity was marked on the patient’s skin at the time of US to determine the field borders. US was performed using a 12-5 MHz linear-array transducer (HDI 5000, Advanced Technology Laboratories, Bothell, WA, USA) by a radiologist. Radiotherapy consisted of treatment to the entire breast with medial and lateral tangential fields to a total dose of 50 Gy in 2 Gy per fraction with a 6MV linear accelerator. The boost field was irradiated by electrons between 10 and 16 Gy.

**Figure 1:**
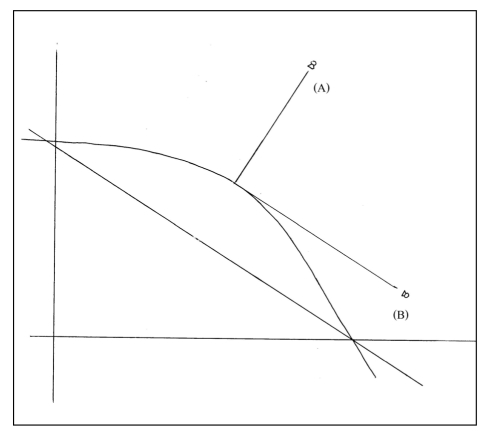
Diagram of electron beam direction (A) Treated direction, (B) 90 degree from treated direction.

At the time of initial radiation therapy simulation of tangential fields, a lead wire was placed over the lumpectomy scar and further films were undertaken for boost planning. The techniques used were as described by Rabinovitch et al. [11]. Orthogonal films were taken with the isocentre at the middle of the scar. The first film was taken with the beam perpendicular to the skin surface at the surgical scar (treatment direction) (Figure 1A, 2A). By using SSD (source-skin distance) technique, the second film was taken 90 degrees from the first, with the beam tangential to the skin surface (Figure 1B, 2B). These films required a couch, gantry and collimator rotation to achieve the desired beam angles. The depth (D_2_) from the skin to the anterior border of the chest wall muscle was measured from the second film. In the absence of clip placement, we could not measure the transverse and longitudinal distances using the radiographic film. Electron energy was chosen by defining “treated depth” (TD) as the higher value between the depth to pectoralis muscle from radiographic film (D_2_), and ultrasound cavity depth (D_1_).

**Figure 2:**
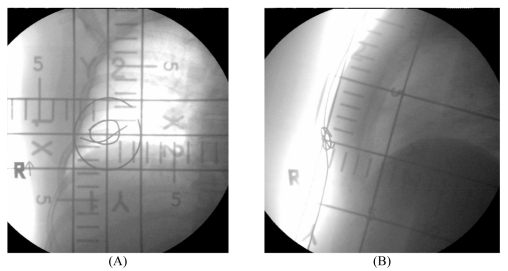
Orthogonal radiographic film of (A) Treated direction, (B) 90 degree from treated direction.

The depth measurements from US and radiographic film were compared using paired t-test. Correlation with the depth measurements from US and radiographic film were determined by the Pearson’s test.

## RESULTS

Forty-one patients were treated in this study. Patients and tumour characteristics are listed in Table 1. The median age was 45 years (range: 27-58). The majority of tumours were between 2.1 and 3.0 cm in diameter. Most patients (87.8%) received chemotherapy before RT. The median interval between surgery and US was 24 weeks (range: 1 - 48). Table 2 gives the mean of the measured depth results for D_1_ and D_2_ and the mean of the absolute difference in depths (i.e. absolute mean of D_2_ – D_1_). The absolute mean difference of the depth (radiographic films minus US) was 0.129 cm. The absolute depth discrepancy between the two methods ranged from 0 to 0.5 cm. A paired t-test demonstrated no statistically significant difference between US and plain film measurements (p=0.27). The Pearson’s correlation coefficients between depth dimensions estimated by US and by radiographic film was 0.98 (n = 41). (repeated)

**Table 1:** Patients and tumor characteristics

	No. of patients	%
Number of patients	41	
Median age	45 (27 - 58)	
Pathological tumor size (cm)		
≤ 1.0	5	12.2
1.1 - 2.0	14	34.1
2.1 - 3.0	15	36.7
3.1 - 4.0	4	9.7
4.0 - 5.0	3	7.3
Chemotherapy regimen		
CMF ՠ6	12	29.3
AC/ EC ՠ4	4	9.7
FAC/ FEC ՠ6	20	48.8
No chemotherapy	5	12.2
Interval from surgery to performing US (weeks)		
≤ 12	11	26.3
13 - 24	10	23.8
25 - 36	16	38
37 - 48	4	9.5

**Table 2:** Comparison of the depth by two methods

Mean depth by US (cm)	2.33
Mean depth by radiographic films (cm)	2.37
p-value (95% CI of the difference)	0.27 (-0.103 - 0.03)
Discrepancy range for depth between two methods (cm)	0 - 0.5

For this study, the chosen electron energy was based on the deepest extent (regardless of method of measurement: US or orthogonal film) and the field borders were defined by marking the extent of the lumpectomy cavity on the patient’s skin at the time of US: i.e. information from both the plane radiographic film and US was used in the planning of the radiation boost treatment. In two patients whose intervals from surgery to US were more than 40 weeks, US evaluation was of no value due to lack of identifiable cavity. Median follow-up time was 47 months (range: 30- 52). Two of the 41 patients (4.9%) developed a local recurrence of the breast cancer outside the boost area at 25 months and 27 months, respectively, after completion of radiotherapy. One of them also developed lung metastases and eventually died. At the time of analysis, 40 patients were still alive with no evidence of disease.

## DISCUSSION

Although CT-based planning with surgical clips remains the most accurate method to delineate the tumour bed, this is not possible with all patients: some do not have surgical clips placed, and some radiation facilities do not have access to CT for this planning. In the absence of surgical clips, Rabinovitch et al. [11] recommended using CT-guided treatment planning. Smitt et al. [5] published information on the use of CT in the absence of surgical clips to visualize the lumpectomy cavity and found results similar to those obtained with US. Conversely where equipment is limited, several studies have found that fluoroscopy of surgical clips is a fairly precise method of delineating the surgical cavity [2, 4, 8, 11, 13].

This study considered the options for patients without surgical clips and where CT is not available. Previous studies [13, 14] have demonstrated successful results of using US to localize the lumpectomy cavity and facilitate boost field placement in patients treated with lumpectomy and radiation therapy but issues can arise with US planning alone. The optimal time to perform US is not known. It should not be performed too early following lumpectomy because significant seroma fluid or haematoma may exist within the cavity, distorting the volume. And it should not be performed too late, such as after the end of chemotherapy cycles, because a long gap post operatively can cause near complete absorption of seroma and lumpectomy cavity and US may not be able to estimate the dimensions of the surgical bed or even discern any cavity. Almost all the patients received chemotherapy before RT except for 5 patients with tumours of ≤ 1 cm. The common chemotherapy regimens were FAC or FEC and half of the patients had cycles delayed due to grade 3 or 4 neutropenia, accounting for the long median time between surgery and RT. Two patients (4.8%) had no identifiable cavity and both of them had a long interval from surgery to US procedure (40 and 48 weeks, respectively). Because of this obscuration of the lumpectomy cavity with time, an increasing gap between surgery and radiotherapy simulation can lead to underestimation of the tumour bed and margins by US [6,11].

In this study, no patients had surgical clips, so radiography could only be used to estimate the depth of the surgical tumour bed (by measuring the depth from the surface to the anterior border of the chest wall) and not the transverse and longitudinal dimensions of the tumour bed. Radiography alone is therefore inadequate for patients without surgical clips. However, as discussed above, it is not clear that the cavity identified by US will still be accurate, or even visualisable, if several months pass before radiation therapy is commenced.

**Figure 3:**
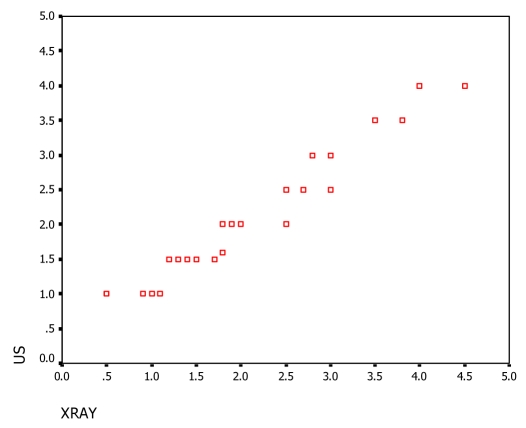
Correlation coefficients between the two measurement methods.

In this study, depth measurements were obtained both from the plane radiograph and from the US. A paired t-test demonstrated no statistically significant difference between the two methods. Neither was consistently deeper than the other. In figure 3, the correlation between both measurements was shown that there are correlation coefficients between them. To avoid geographical miss, the safest approach is therefore to integrate both methods and use the deepest of the two depth measurements to define the electron energy and the US to delineate the radiation field margins.

Although CT-based planning with surgical clips remains the most accurate method to delineate tumour bed, fluoroscopy in combination with US is also a fairly acceptable technique for boost field delineation. The authors would encourage breast surgeons to place surgical clips in the walls of the lumpectomy cavity, so that further studies can be undertaken to compare the transverse and longitudinal dimensions as determined by fluoroscopy and US, and to confirm the effectiveness of this integrated technique.

## References

[1] Pierce LJ, Moughan J, White J (2005). 1998-1999 patterns of care study process survey of national practice patterns using breast-conserving surgery and radiotherapy in the management of stage I-II breast cancer. Int J Radiat Oncol Biol Phys.

[2] Bedwinek J (1993). Breast conserving surgery and irradiation: the importance of demarcating the excision cavity with surgical clips. Int J Radiat Oncol Biol Phys.

[3] Hunter MA, McFall TA, Hehr KA (1996). Breast-conserving surgery for primary breast cancer: necessity for surgical clips to define the tumor bed for radiation planning. Radiology.

[4] Harrington KJ, Harrison M, Bayle P (1996). Surgical clips in planning the electron boost in breast cancer: a qualitative and quantitative evaluation. Int J Radiat Oncol Biol Phys.

[5] Birdwell RL, Ikeda DM, Torrey MJ (1997). Sonographic tailoring of electron beam boost site after lumpectomy and radiation therapy for breast cancer. AJR Am J Roentgenol.

[6] DeBiose DA, Horwitz EM, Martinez AA (1997). The use of ultrasonography in the localization of the lumpectomy cavity for interstitial brachytherapy of the breast. Int J Radiat Oncol Biol Phys.

[7] Gilligan D, Hendry JA, Yarnold JR (1994). The use of ultrasound to measure breast thickness to select electron energies for breast boost radiotherapy. Radiother Oncol.

[8] Kovner F, Agay R, Merimsky O (1999). Clips and scar as the guidelines for breast radiation boost after lumpectomy. Eur J Surg Oncol.

[9] Machtay M, Lanciano R, Hoffman J (1994). Inaccuracies in using the lumpectomy scar for planning electron boosts in primary breast carcinoma. Int J Radiat Oncol Biol Phys.

[10] Sedlmayer F, Rahim HB, Kogelnik HD (1996). Quality assurance in breast cancer brachytherapy: geographic miss in the interstitial boost treatment of the tumor bed. Int J Radiat Oncol Biol Phys.

[11] Rabinovitch R, Finlayson C, Pan Z (2000). Radiographic evaluation of surgical clips is better than ultrasound for defining the lumpectomy cavity in breast boost treatment planning: a prospective clinical study. Int J Radiat Oncol Biol Phys.

[12] Smitt MC, Birdwell RL, Goffinet DR (2001). Breast electron boost planning: comparison of CT and US. Radiology.

[13] Denham JW, Sillar RW, Clarke D (1991). Boost dosage to the excision site following conservative surgery for breast cancer: it's easy to miss. Clin Oncol (R Coll Radiol).

[14] Helyer SJ, Moskovic E, Ashley S (1999). A study testing the routine use of ultrasound measurements when selecting the electron energy for breast boost radiotherapy. Clin Oncol (R Coll Radiol).

[15] Lemanski C, Azria D, Thezenas S (2006). Intraoperative radiotherapy given as a boost for early breast cancer: long-term clinical and cosmetic results. Int J Radiat Oncol Biol Phys.

